# Neuroprotective Effects of Low-Dose Statins in the Retinal Ultrastructure of Hypercholesterolemic Rabbits

**DOI:** 10.1371/journal.pone.0154800

**Published:** 2016-05-04

**Authors:** Judith Fernández-Navarro, Pilar Aldea, Rosa de Hoz, Juan J Salazar, Ana I Ramírez, Blanca Rojas, Beatriz I. Gallego, Alberto Triviño, Teresa Tejerina, José M. Ramírez

**Affiliations:** 1 Instituto de Investigaciones Oftalmológicas Ramón Castroviejo. Universidad Complutense Madrid (UCM), Spain; 2 Facultad de Óptica y Optometría, UCM, Spain; 3 Facultad de Medicina, UCM, Spain; 4 Department of Pharmacology, School of Medicine, Complutense University, Madrid, Spain; Children's Hospital Boston, UNITED STATES

## Abstract

To evaluate the pleiotropic effects to statins, we analyze the qualitative and quantitative retinal changes in hypercholesterolemic rabbits after a low-dosage statin treatment. For this purpose, New Zealand rabbits were split into three groups: control (G0; n = 10), fed a standard diet; hypercholesterolemic (G1; n = 8), fed a 0.5% cholesterol-enriched diet for 8 months; and statins (G2; n = 8), fed a 0.5% cholesterol-enriched diet for 8 months, together with the administration of statin (pravastatin or fluvastatin sodium) at a dose of 2 mg / kg / day each diet. The retinas were analyzed by transmission electron microscopy and immunohistochemistry (glial fibrillary acidic protein). The retinal thickness of nuclear and plexiform layers were quantified in semi-thin sections. The results revealed that the low-statin-treated rabbits in comparison with the hypercholesterolemic group showed: i) a more preserved structure in all retinal layers; ii) a significant reduction in retinal thickness; iii) a decrease in cell death in the nuclear-and ganglion-cell layers; iv) a reduction of hydropic degeneration in the plexiform and nerve-fiber layers; v) a preservation of astrocytes and of the retinal area occupied by them; and vi) a better-preserved retinal vascular structure. Our findings indicate that low doses of statins can prevent retinal degeneration, acting on retinal macroglia, neurons and retinal vessels, despite that hypercholesterolemia remained unchanged. Thus, the pleiotropic effects of the statins may help safeguard the retinal ultrastructure.

## Introduction

Hypercholesterolemia constitutes an ischemic and inflammatory risk factor in certain neurodegenerative diseases [1]. Cholesterol, the main sterol in the retina, is widely distributed in all cell layers of the neuroretina, including the ganglion-cell layer and the nerve-fiber layer. The retina acquires cholesterol both by endogenous synthesis and by circulating lipoproteins [2]. The retina is capable of rapid uptake of circulating LDL via an LDLR-mediated process occurring primarily in the RPE and also possibly in Müller cells [3]. Under dietary influence and *in situ* synthesis, cholesterol metabolism is regulated by cell interactions, including neurons and glial cells. Glia is an active partner of neurons, maintaining cholesterol synthesis and removal [1]. Glial-derived cholesterol has been implicated as an important source of neuronal cholesterol, particularly for making new synaptic membranes [4]. Dysregulation of cholesterol in the retina has been associated with retinal impairment, reflecting the key role of this sterol in neurons [1]. Cellular cholesterol deficiency and accumulation, hallmarks of some neurodegenerative diseases involving the retina [5,6], highlight the importance of maintaining cholesterol homeostasis in neurons. Many genes historically associated with liver, intestine, and adipose cholesterol homeostasis are expressed in retina, RPE and choroid [7,8]. Notably, the association of several genes involved in lipid metabolism with age-related macular degeneration (AMD) has been recently described [9]. With aging, apolipoprotein B-lipoprotein particles of unusual composition are secreted by RPE and accumulate in Bruch’s membrane forming a lipid wall, a precursor of the basal linear deposits seen in AMD [10]. Esterified and non-esterified cholesterol can constitute at least 40% of drusen [11,12], which are AMD-specific lesions.

Hypercholesterolemia reportedly boosts nitric oxide synthase 2 expression in the retina, causing lipid peroxidation and thereby leading to oxidative damage in tissues [13]. Experimental studies have demonstrated that a cholesterol-enriched diet not only induces ultrastructural changes in the retinal macroglia, in cells of the outer and inner retina and in endothelial cells of the retinal capillaries but also increases lipids in the retinal pigment epithelium (RPE) and triggers changes in Bruch’s membrane and the choroid [14–18]. These hypercholesterolemia-related changes, secondary to chronic ischemia in the retina, resemble those found in human AMD [19,20].

Neuroprotection, the therapeutic paradigm designed to slow or prevent the death of neurons in order to maintain physiological function has long been a goal of clinical and basic neuroscience when treating neurodegeneration in the brain and in the eye [21]. Some researchers have associated 3-hydroxy-3 methylglutaryl coenzyme A reductase inhibitors or “statins” with a reduced risk of dementia, depression [22] and open-angle glaucoma [23]. However, information regarding statin trials in AMD are inconclusive [24,25]. Statins are prescribed to help regulate low-density lipoprotein-cholesterol levels [26]. However, it is now recognized that statins also have pleiotropic effects that activate a general neuroprotective mechanism. That is, statins may mitigate oxidative stress, stabilize the atherosclerotic plaque, restore endothelial function, reduce blood-vessel inflammation [26–30], and favor the expression of neuroprotective genes in the brain [31]. In rabbits fed a cholesterol diet, these pleiotropic effects have resulted from statin doses insufficient to reduce plasma-cholesterol levels. [27,32]. In addition, we have previously demonstrated in hypercholesterolemic New Zealand rabbits that statin treatment at non-lipid-lowering doses can prevent the progression of atherosclerotic ischemia in the different vascular layers of the choroid [33]. As a second step of the aforementioned study, the aim of the present work was to analyze whether besides the effects on the choroid, a dose of statins insufficient to normalize plasma-lipid levels could improve retinal protection by acting on retinal macroglia and neurons.

## Material and Methods

### Experimental Design

Twenty-six adult male New Zealand rabbits weighing 2.5 ± 0.5 Kg were caged separately in an air-conditioned room with a 12-h light/dark cycle. All animals were fed a rabbit-maintenance diet (carbohydrates [N.F.E.] 50%, fiber 15.5%, protein 13.5%, moisture 11%, minerals 7%, and lipids 3%; Panlab S.L. Barcelona, Spain) at least 7 days before the beginning of the experiment and allowed *ad libitum* access to water. The rabbits were assigned to three groups: control (G0; n = 10), fed a rabbit maintenance diet for 8 months; hypercholesterolemic rabbits (G1; n = 8), fed a rabbit maintenance diet enriched with 0.5% cholesterol (U.A.R., Paris, France) for 8 months; statin (G2; n = 8), fed a rabbit standard diet enriched with 0.5% cholesterol (U.A.R., Paris, France) plus the administration of fluvastatin sodium (a entirely synthetic lipophilic statin metabolized in the liver) (Novartis) at a dose of 2 mg/Kg/day (G2A; n = 4) or pravastatin sodium (a hydrophilic statin of fungal origin) (Bristol-Myers Squibb) at a dose of 2 mg/Kg/day (G2B; n = 4) for 8 months. Two rabbits from the hypercholesterolemic group (G1) and one rabbit from the pravastatin group (G2A) died as a result of cutaneous infection during the course of the experiment.

Diet consumption was checked daily and weight was recorded at the beginning of the experiment and monitored monthly thereafter. The same schedule was used to monitor serum values of total cholesterol. For this, blood samples were taken from the marginal vein of the ear and analyzed by a colorimetric reaction using a commercially available kit (BioMerieux, France). The animals were handled following institutional guidelines, European Union regulations for animal use in research, and the ARVO (Association for Research in Vision and Ophthalmology) statement for the use of animals in ophthalmic and vision research, and approval by the ethical committee of Complutense University (Spain).

The rabbits were killed with an overdose of sodium pentobarbital. Eyes were enucleated immediately after death and slit behind the limbus with a razor blade in order to facilitate penetration of the fixative. For each animal, one of the eyes was used for immunohistochemistry (n = 23) and the other one for electron microscopy (n = 23).

### Transmission Electron Microscopy (TEM)

Eyes were immersed in 2% glutaraldehyde in 0.1M phosphate buffer (PB), at pH 7.4 and 4°C for 5h. After being washed in 0.1M PB, the wall of the posterior segment of the eyes (including the choroid, RPE, and neurosensorial retina) was diced into small pieces. These fragments were post-fixed in 1% osmium tetroxide in 0.1M PB for 2h at 4°C. The tissues were then dehydrated in graded acetone and embedded in araldite. The semi-thin sections (0.5μm) were stained with toluidine blue, and after selection the blocks were further trimmed for ultramicrotomy (Reichert OM-V3 ultramicrotome, Leica, Germany). The thin sections, treated with 2% uranyl acetate in water and lead citrate for contrast, were examined by transmission electron microscopy (TEM; Zeiss 902 electron microscope) to study the RPE and the neurosensory retina.

### Immunohistochemistry

To analyze the retinal macroglia, we fixed eyes with 4% paraformaldehyde in 0.1M PB (pH 7.4) for 4h and then processed as retinal whole-mounts with the immunohistochemical protocol described elsewhere [34]. A monoclonal antibody directed against glial fibrillary acidic protein (GFAP) (clone GA-5, Sigma, St. Louis, MO, USA) was used as a primary antibody in a 1/300 dilution. A negative control was performed to demonstrate that the secondary antibody reacted only with its respective primary antibody. This control was made by eliminating the primary antibody and replacing it with antibody buffer.

### Quantification of Retinal-Layer Thickness

Quantitative analyses were made in six rabbits from each study group (G0, G1, G2). For each animal, we measured six retinal semi-thin sections of the same retinal zones (medulated nerve-fiber region: MNFR). On each section, measurements in three non-adjacent retinal areas were performed as described elsewhere [33]. Photographs were taken at 40X with a microscope (Zeiss, Axioplan 2 Imaging Microscope). In each semi-thin section photographed, a longitudinal zone of 150μm was selected to quantify retinal-layer thickness.

The retinal thickness was measured in the following layers of the neurosensory retina: photoreceptor layer (PL), outer nuclear layer (ONL), outer plexiform layer (OPL), inner nuclear layer (INL), and inner plexiform layer (IPL). The ganglion cell layer and the nerve fiber layer were not included in the measurements, since the retinal zone for the quantification was the medulated nerve-fiber region (MNFR) in which the astrocytes are located. In the rabbit, this zone is a vascularized retinal region where axons are myelinated, causing great thickness variability. The retinal thickness was manually measured using an interactive measurement tool included in the AxioVision Release 4.8.2 computer program (Zeiss, Germany) coupled to the microscope.

### Quantitative Analysis of Retinal Astrocytes

The area occupied by astrocytes associated with the nerve-fiber bundles (AANFB) and that occupied by perivascular astrocytes (PVA) were quantified in the retinal whole-mounts as described elsewhere [17].

#### GFAP-labeled retinal area occupied by astrocytes associated with the nerve-fiber bundles (AANFB-RA)

Briefly, the GFAP-labeled retinal area occupied by AANFB was measured in previously selected zones. The selection criterion was that the zones should be free of perivascular astrocytes, given that their presence could distort or hamper the AANFB estimation. In each of the MNFR, 9 zones were selected and photographed with a 20X microscopic lens, giving an area of 0.1889 mm^2^ per photograph.

The resulting images were processed with the Threshold Tool of the Metamorph Imaging System. Areas of the image that were marked in the red (as a visual indicator of the thresholded areas) threshold overlay (GFAP+ AANFB) were included in the measurement and processing.

#### GFAP-labeled retinal area occupied by perivascular astrocytes (PVA-RA)

In rabbits, perivascular astrocytes (PVA) took two shapes as described elsewhere [16,17,35,36]:

1. The type I PVA had an ovoid perikaryon, producing numerous sprouting, hair-like processes. These cells are associated with medium-sized epiretinal vessels, and with capillaries located over the inner limiting membrane.
2. The type II PVA, star-shaped, had a spherical cell body with four to ten small radial processes protruding from it which made contact with the vessel wall. These cells are found on larger and medium-sized epiretinal vessels.

The GFAP+ retinal area occupied by type I PVA (PVA-I) and type II PVA (PVA-II) was quantified as previously described for AANFB with slight modifications: i) we selected 13 zones in the centermost part of the MNFR, where the perivascular astrocytes were located; and ii) as the areas of the image marked with the threshold overlay corresponded to the GFAP+ astrocytes (PVA plus AANFB), to quantify just the area containing PVA-I and PVA-II, we then selected these cells by hand using the interactive-mode tool of the Metamorph Imaging System, which allows the selection of individual objects by clicking on them in the image window.

### Statistical Analysis

Data for the statistical analyses were introduced and processed in a SPSS 15.0 (comprehensive statistical software; SPSS Inc). The Mann—Whitney U test was used to analyze the data of weights and serum-lipid values. For retinal thickness, AANFB-RA and PVA-RA differences in means were analyzed using an ANOVA and a *post hoc* Bonferroni test. A value of p < 0.05 was considered statistically significant.

## Results

No statistically significant differences were found in weights and total serum cholesterol levels between the untreated rabbits and those treated with statins (Table 1).

**Table 1:** Weights, total cholesterol serum values at the end of the experiment.

	Group	Mean±SEM	p*
**Weight (Kg)**	G1	3.920 ± 0.38	NS
	G2	3.957 ± 0.28	NS
**Cholesterol (mg/100ml)**	G1	1061 ± 358	NS
	G2	968 ± 162	NS

* Mann—Whitney test. NS = Non-significant; G1 (n = 8) (0.5% cholesterol-enriched diet. No treatment); G2 (n = 8) [0.5% cholesterol-enriched diet + fluvastatin sodium or pravastatin sodium (2 mg/Kg/day each)].

Since no substantial differences appeared in morphology or ultrastructural features of the retina between statin groups, the results of G2A and G2B groups are described together as G2.

### Semi-Thin Sections (Light Microscopy)

**Fig 1:**
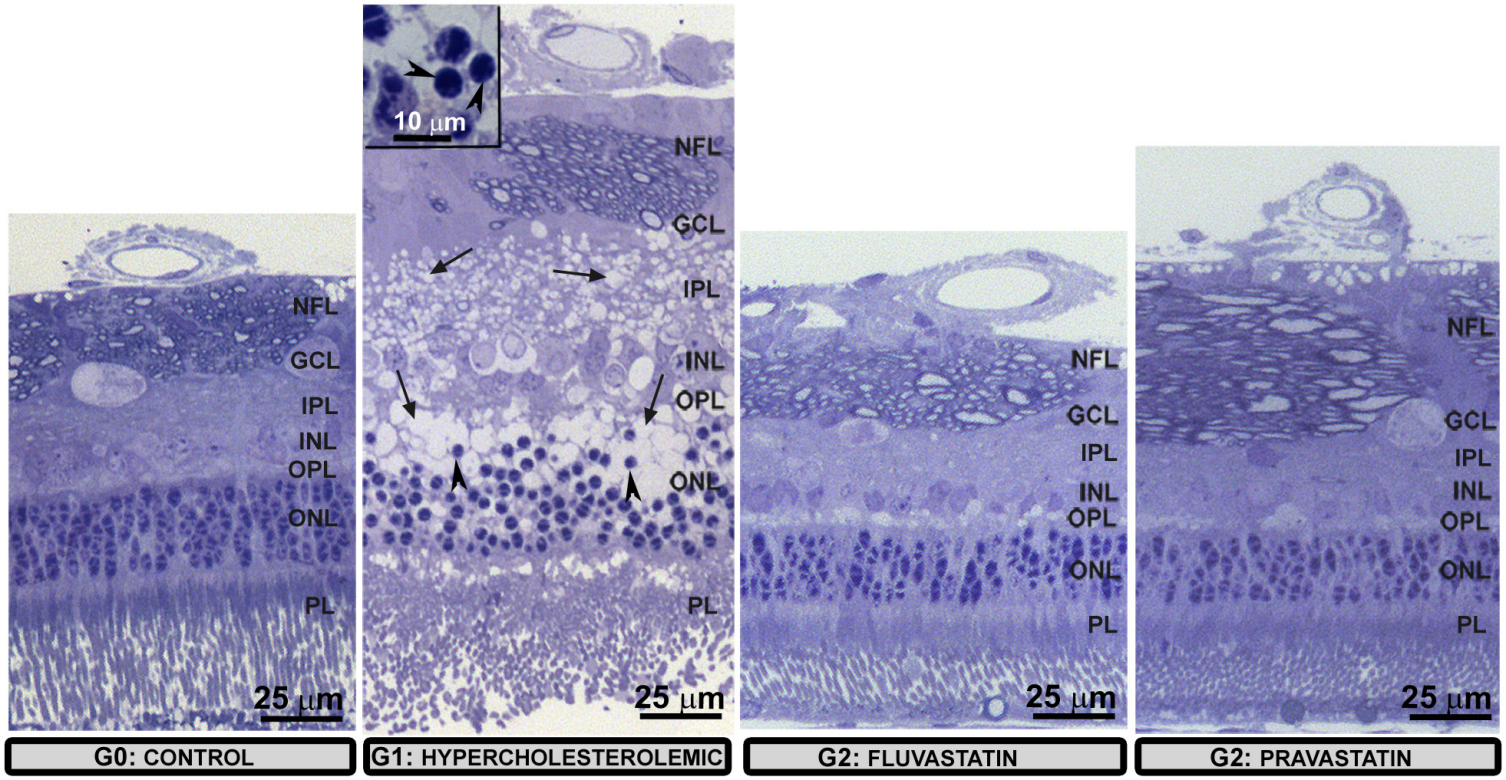
Semi-thin sections (optic microscopy). In G1 the figure illustrates the overall alterations of the retinal layers: pyknotic nuclei (arrowheads) and edema (arrows) secondary to cell degeneration. Details of pyknotic nuclei (inset). The retina in G2 was more similar to G0; [ganglion-cell layer (GCL); inner nuclear layer (INL); inner plexiform layer (IPL); nerve-fiber layer (NFL); outer nuclear layer (ONL); outer plexiform layer (OPL); photoreceptor layer (PL)].

Overall, in comparison to G0 and G2, G1 rabbits exhibited several alterations in all retinal layers. In G1, retinal layers were disorganized, showing edema, empty spaces, and pyknotic nuclei, which were not detected in G2, the latter being more similar to the G0 group (Fig 1).

### Quantification of the Retinal-Layer Thickness

**Fig 2:**
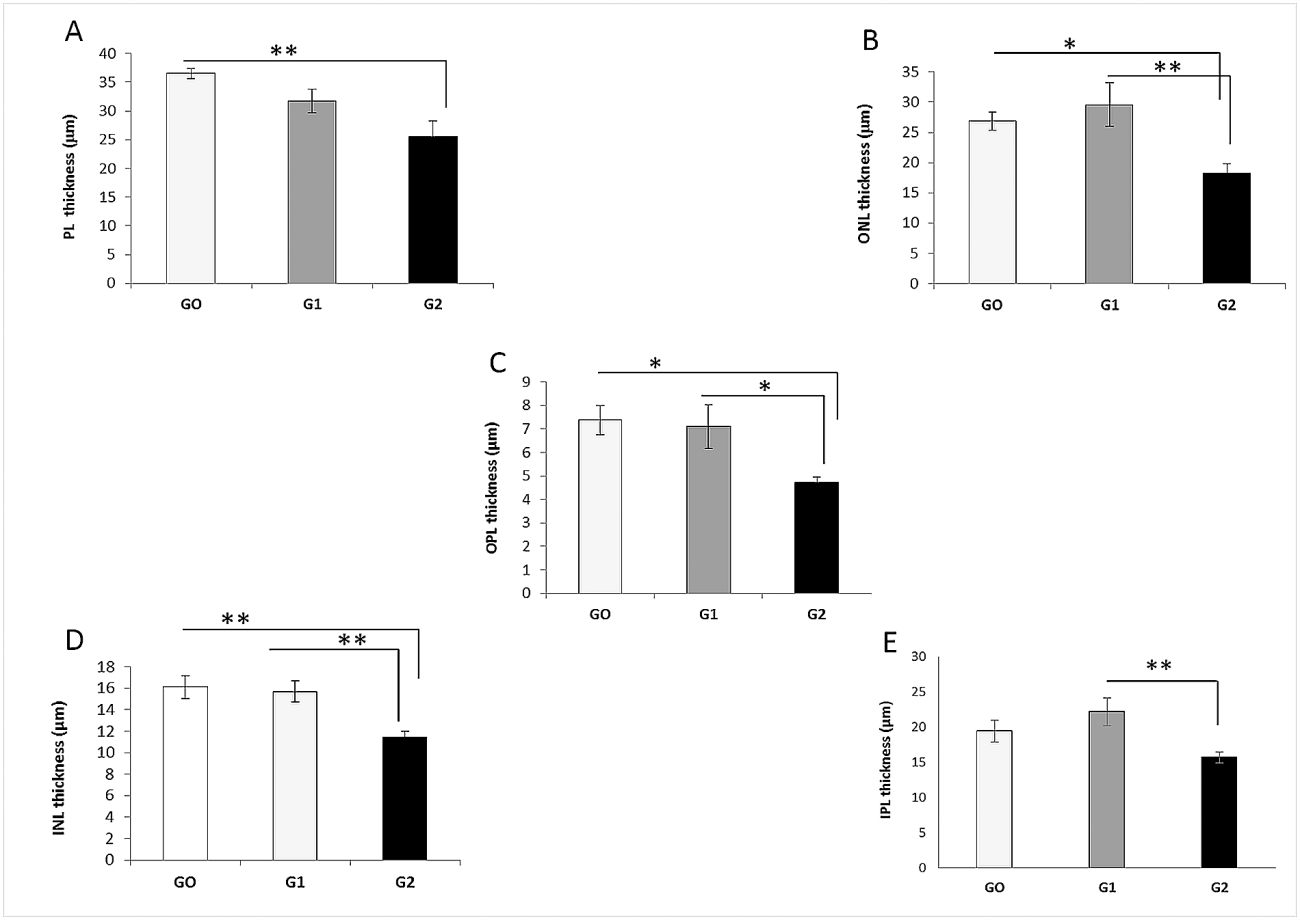
Retinal-layer thickness. **Comparison among the three groups of study.** A: The photoreceptor layer was significantly thinner in G2 than in G0. ** p<0.01 vs. G0; B: The outer nuclear layer was significantly thinner in G2 than in G0 and in G1. ** p<0.01 vs. G1; * p<0.05 vs. G0. C: The outer plexiform layer was significantly thinner in G2 than in G0 and in G1. * p<0.05 vs. G1; * p<0.05 vs. G0. D: The inner nuclear layer was significantly thinner in G2 than in G0 and in G1. ** p<0.01 vs. G1; ** p<0.01 vs. G0; E: The inner plexiform layer was significantly thinner in G2 than in G1. ** p<0.01 vs. G1; Each bar represents the mean ± SD. ANOVA with Bonferroni test. ANOVA, analysis of variance. [INL: inner nuclear layer; IPL: inner plexiform layer; ONL: outer nuclear layer; OPL: outer plexiform layer; PL: photoreceptor layer].

In G2, the thickness of the different retinal layers significantly diminished in comparison both to G1 (p < 0.01 for ONL; p < 0.05 for OPL; p < 0.01 for INL; p < 0.01 for IPL, ANOVA in all cases) and to G0 (p < 0.01 for PL; p < 0.05 for ONL; p < 0.05 for OPL; p < 0.01 for INL, ANOVA in all cases) (Fig 2).

### Transmission Electron Microscopy

#### Retinal-pigment epithelium

**Fig 3:**
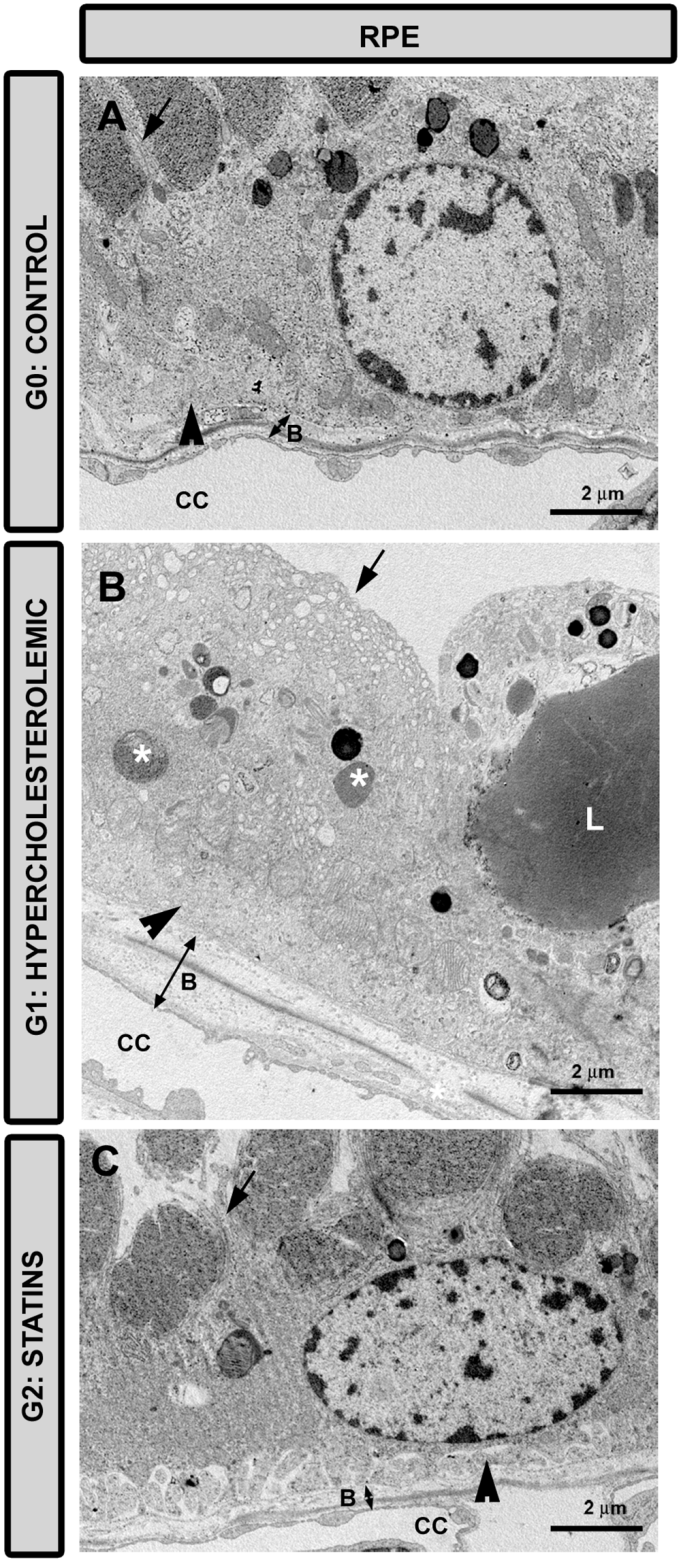
Transmission electron microscopy of the RPE cells. A: G0 control; B: G1 hypercholesterolemic; C: G2 statins. The cytoplasm of RPE cell in G1 (B) shows dense bodies (*) and droplets of lipids (L) that were less evident in G2 (C). Apical microvilli (arrow) and basal infolding (arrowhead) were better preserved in G2 (C) than in G1 (B). [Bruch’s membrane (B); choriocapillaris (CC). G2 (Pravastatin)].

In G1 the retinal-pigment epithelium (RPE) cells were hypertrophic (Fig 3B). Their cytoplasm showed numerous dense bodies and many lipid droplets. These abnormalities appeared to a much lesser extent in G2 (Fig 3C). Also, basal infoldings and microvilli were better preserved in G2 (Fig 3C) than in G1 (Fig 3B), where these structures disappeared in some retinal sectors.

#### Photoreceptor Layer

**Fig 4:**
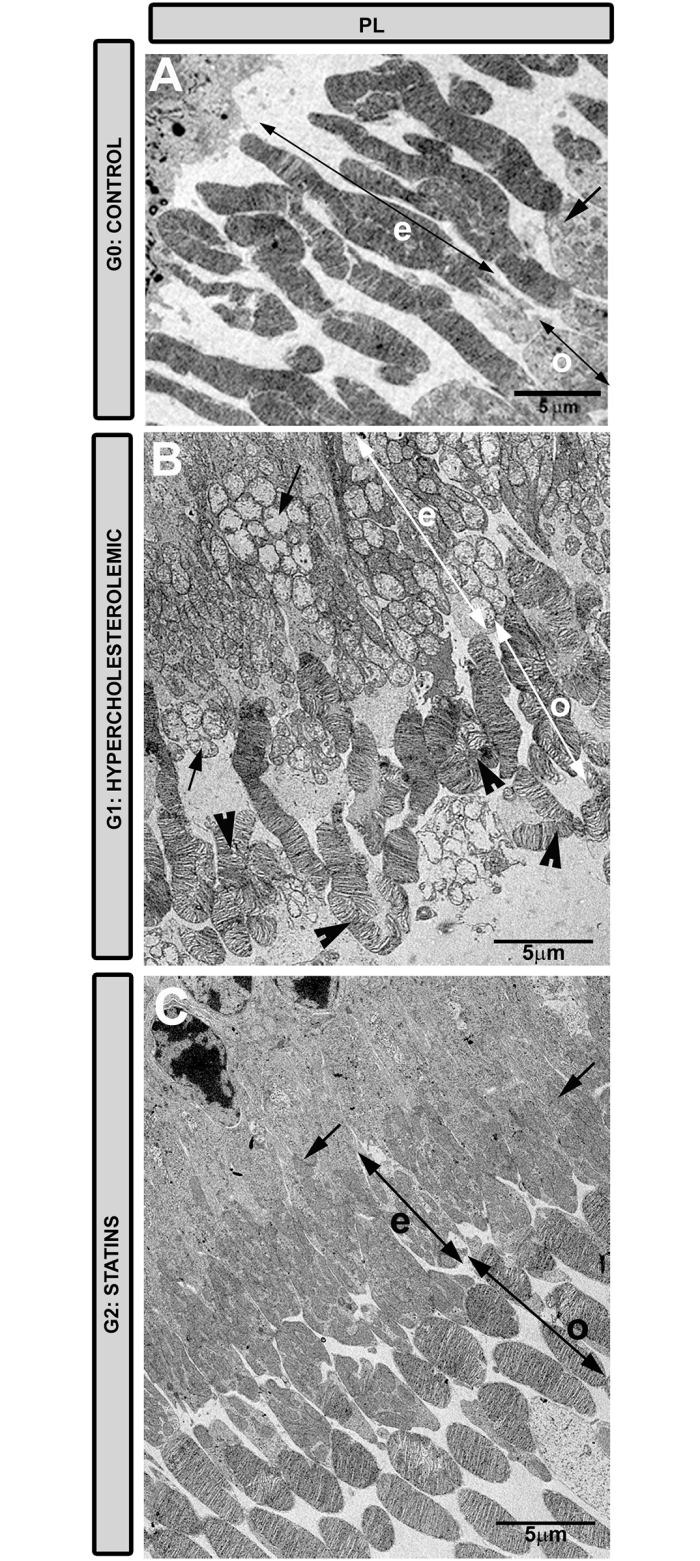
Transmission electron microscopy of the photoreceptor layer. A: G0 control; B: G1 hypercholesterolemic; C: G2 statins. In G1 (B) the outer segment of the photoreceptors formed circumvolutions (arrowheads) that were not detected in G2 (C). The mitochondria of the ellipsoid were swollen (arrows) in G1 (B). In G2 (C) they were similar to those in G0 (A). [Outer segment of photoreceptors (o); photoreceptor ellipsoid (e). G2 (pravastatin)].

In G1 (Fig 4B) the mitochondria of the photoreceptor ellipsoid were swollen and degenerated, unlike the numerous well-preserved mitochondria detected in G2 (Fig 4C). In G1 the outer segment of the photoreceptors formed circumvolutions (Fig 4B), an alteration rarely found in G2 (Fig 4C).

#### Outer nuclear layer

**Fig 5:**
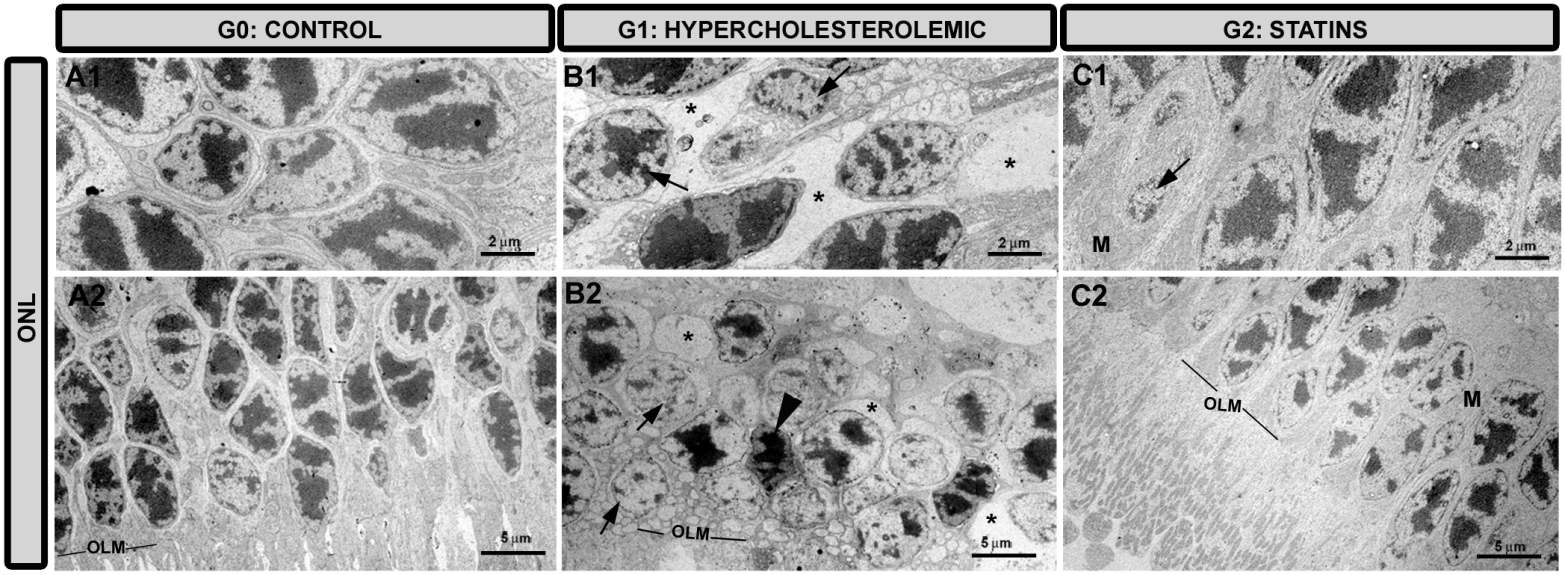
Transmission electron microscopy of the outer nuclear layer. A1,A2: G0 control; B1,B2: G1 hypercholesterolemic; C1,C2: G2 statins. In G1 empty spaces (*), and cell death by necrosis (arrow) and apoptosis (arrowhead) were detected in most photoreceptors. In G2 the signs of cell death were less apparent, being more similar to those in G0. In G2 the spaces left by dead cells were filled by Müller cells. [Müller cells (M); outer limiting membrane (OLM). G2 (pravastatin)].

In G1 (Fig 5B1 and 5B2), most cells were intensely degenerated due to necrosis and apoptosis, and empty spaces appeared between cells. In G2 (Fig 5C1 and 5C2), most cells were well preserved and the empty spaces visible in G1 (Fig 5B1 and 5B2) were occupied by Müller-cell processes (Fig 5C1 and 5C2).

#### Outer plexiform layer

**Fig 6:**
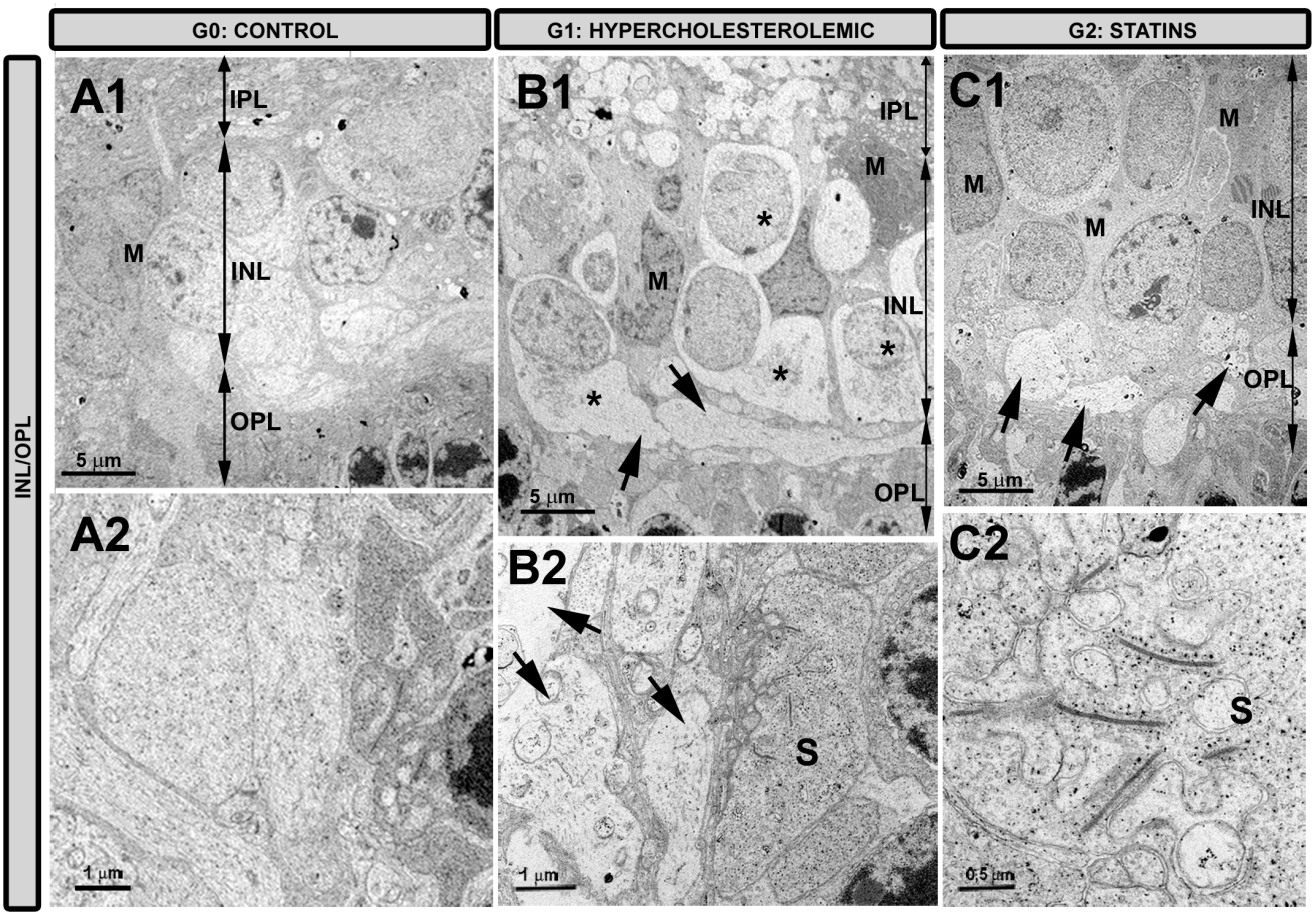
Transmission electron microscopy of the inner nuclear layer and outer plexiform layer. A1,A2: G0 control; B1,B2: G1 hypercholesterolemic; C1,C2: G2 statins. The signs of necrosis in the cell bodies (*), axons, and dendrites (arrows) were less evident in G2 than in G1. The empty spaces left by degenerated cell bodies and processes observed in G1 (B1) were occupied by processes of Müller cells in G2 (C1). The synaptic complexes were better preserved in G2 (C2) than in G1 (B2). [inner nuclear layer (INL); inner plexiform layer (IPL); Müller cells (M); outer nuclear layer (ONL); outer plexiform layer (OPL); synaptic complexes (S). G2C (pravastatin); G2D (fluvastatin)].

In G1 (Fig 6B1 and 6B2), axons and dendrites were swollen, had necrotic features, and were farther apart than in control (Fig 6A1 and 6A2); also, cells contained numerous dense bodies. In G2 (Fig 6C1 and 6C2), alterations were similar to those of G1, but the changes were less intense. The synaptic complexes, although visible in G1 (Fig 6B2), were better preserved in G2, which displayed numerous synaptic vesicles, synaptic ribbon, and arciform densities (Fig 6C2). The empty spaces left by degenerated axons and dendrites (Fig 6A) were occupied by the Müller-cell processes in G2 (Fig 6C1), a feature not detected in G1.

#### Inner nuclear layer

In G1 (Fig 6B1), most cells in this layer were degenerated, with necrotic predominating over apoptotic features. Cell debris from necrotic and apoptotic cells were phagocytosed by Müller glia (Fig 6B1). G2 cells exhibited normal features or incipient stages of necrosis, and the empty spaces left by the latter were occupied by Müller cells (Fig 6C1).

#### Inner plexiform layer

**Fig 7:**
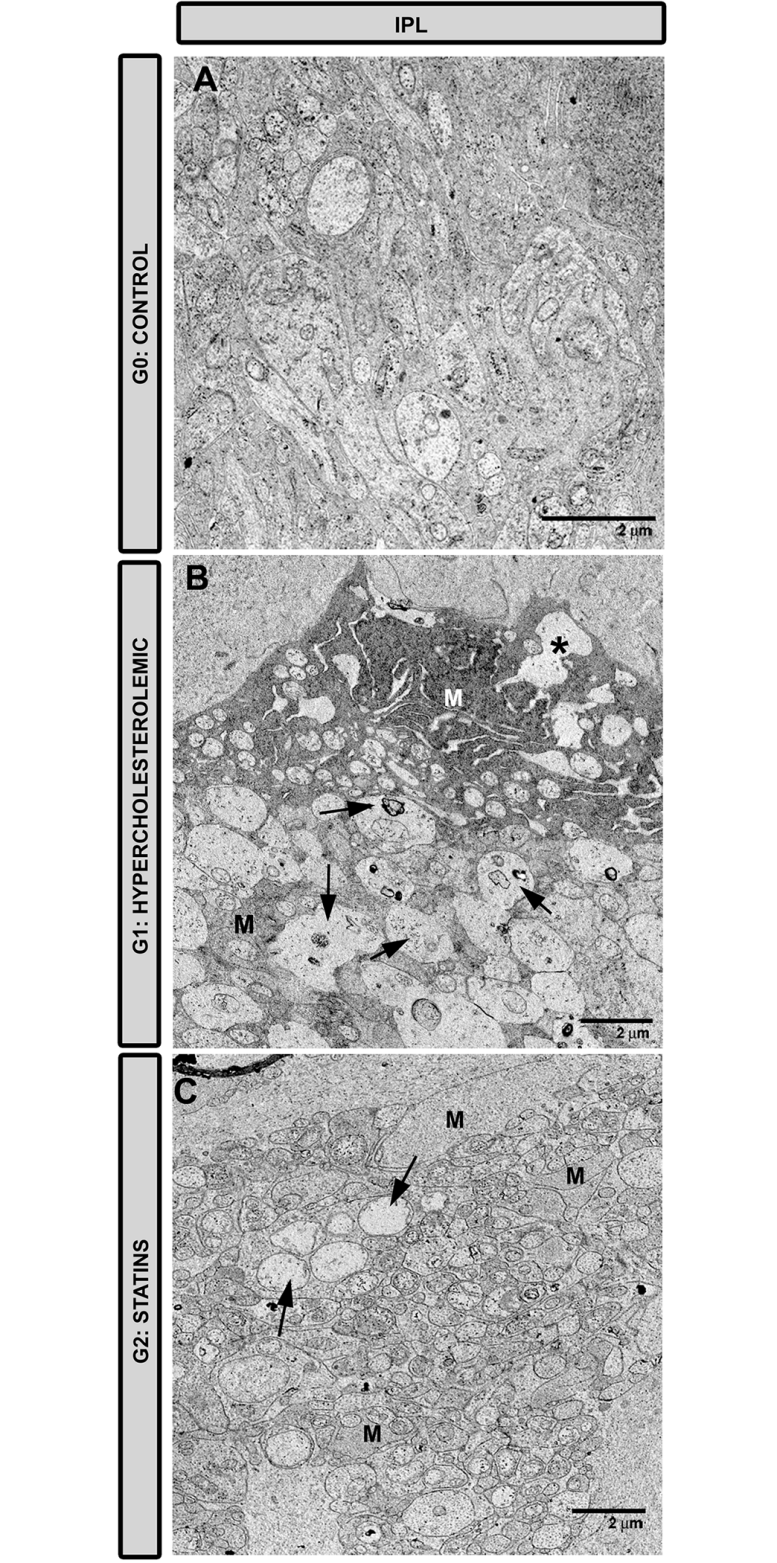
Transmission electron microscopy of the inner plexiform layer. A: G0 control; B: G1 hypercholesterolemic; C: G2 statins. In G2 (C) features of axon and dendrites necrosis (arrows) were fewer than in G1 (B). In G2 (C) the ultrastructural characteristics of the neuronal processes were more similar to those in G0 than in G1 (B) (A). In G2 (C), Müller cells filled the empty spaces left by necrotic cell processes [Müller cells (M); Müller cell in necrosis (*); G2 (pravastatin)].

In G1, axons and dendrites were swollen and exhibited different necrotic features. Mitochondria were swollen and Müller cells were necrotic (Fig 7B). In G2 (Fig 7C), the edematous axons and dendrites appeared less frequently observed than in G1, being more similar to control (Fig 7A). In G2 (Fig 7C), Müller cells filled the empty spaces left by degenerated axons, while neurofilaments and mitochondria were better preserved than in G1 (Fig 7B).

#### Retinal-ganglion-cell layer

**Fig 8:**
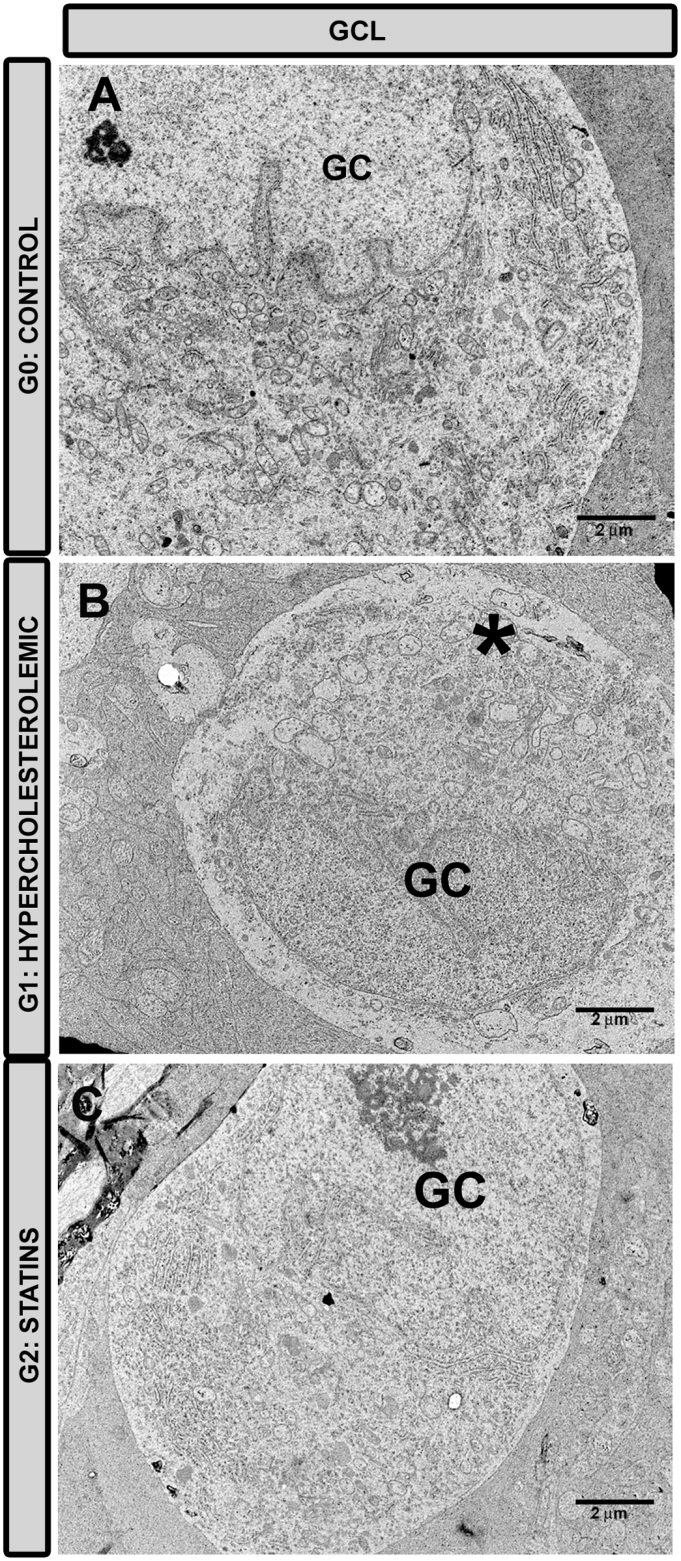
Transmission electron microscopy of ganglion-cell layer. A: G0 control; B: G1 hypercholesterolemic; C: G2 statins. In G1 (B) RGC were necrotic (*); however, in G2 (C) these cells were more similar to those in G0 (A). [Retinal-ganglion cell (GC); G2 (Pravastatin)].

In G1, almost all RGC were necrotic (Fig 8B1), their nucleoplasm, cytoplasm, and cytoplasmic organelles having undergone hydropic degeneration (vacuolization, swelling, and loss of ultrastructural features). However, in G2 (Fig 8C), most of the ganglion cells appeared normal, although some showed incipient necrosis. Apoptosis was not found in the sections analyzed in this retinal layer.

#### Nerve-fiber layer

**Fig 9:**
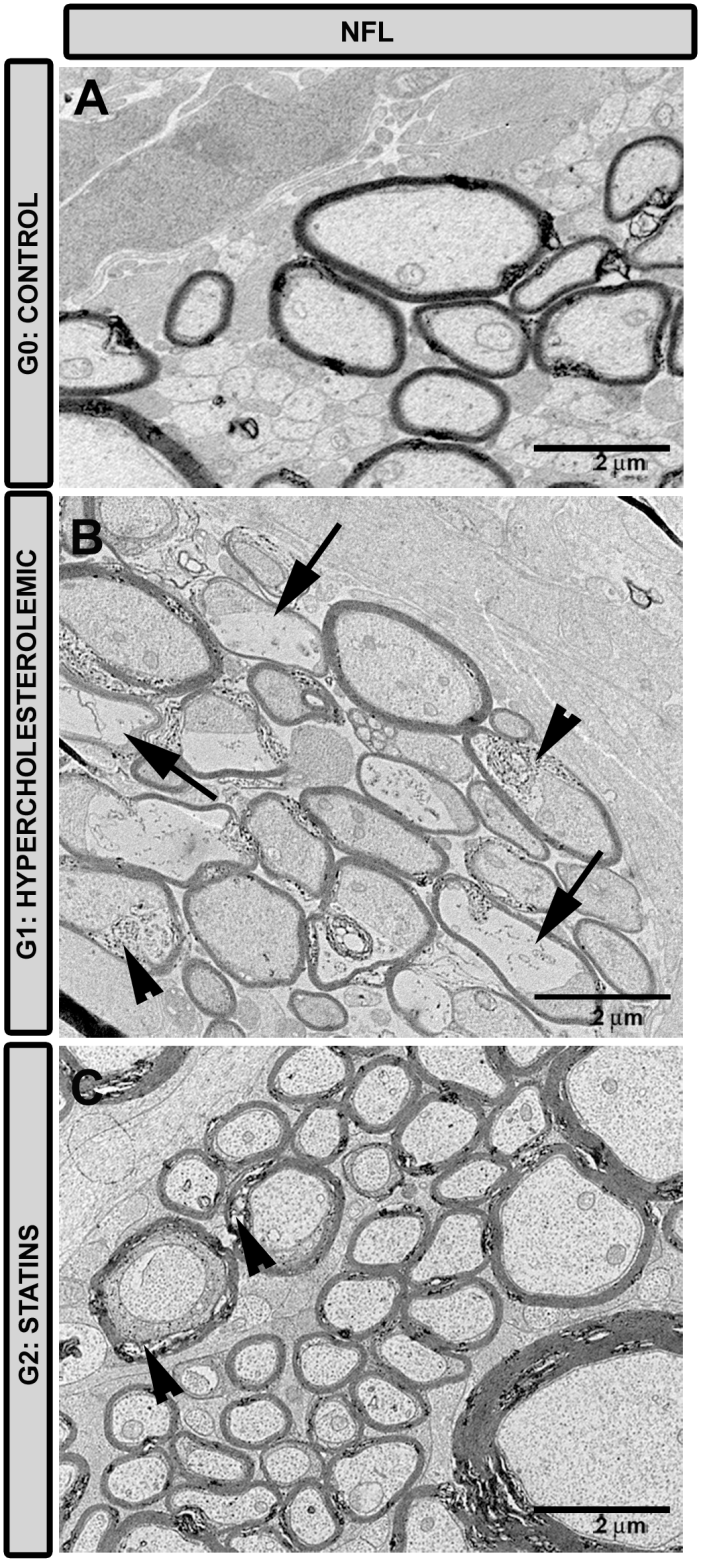
Transmission electron microscopy of nerve-fiber layer. A: G0 control; B: G1 hypercholesterolemic; C: G2 statins. In G2 (C) the ultrastructural characteristic of axons were more preserved than in G1 (B) in which they were necrotic (arrow). In G2 (C) some myelin abnormalities were found (arrowhead) to a lesser extent than in G1 (B). [G2 (fluvastatin)].

This layer showed intense abnormalities in G1 rabbits (Fig 9B). Overall, axons exhibited hydropic degeneration, with neurofilaments and vesicles showing granular disintegration. In some axons, small vacuoles separated the myelin sheaths. In G2 (Fig 9C) axons were better preserved although some myelin abnormalities were visible.

#### Retinal astrocytes and Müller cells

**Fig 10:**
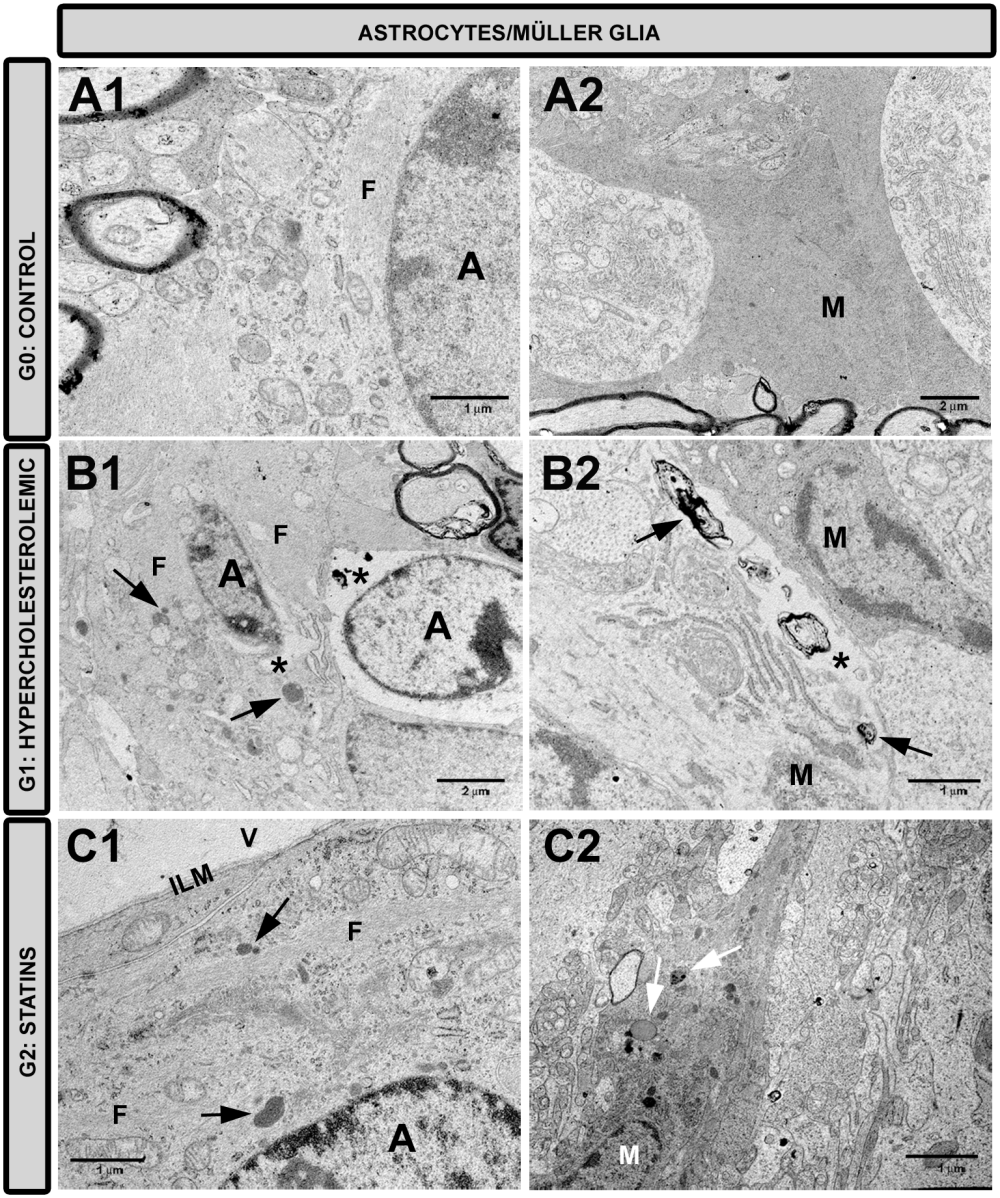
Transmission electron microscopy of retinal macroglia. A1,A2: G0 control; B1,B2: G1 hypercholesterolemic; C1,C2: G2 statins. Astrocytes (A1-C1). Müller cells (A2-C2). In G1 (B1,B2), some astrocytes and Müller cells were necrotic (*) and some others were reactive with abundant organelles, glial filaments, and clumps of electrodense material (arrow). In G2 (C1, C2) these glial cells were reactive. [astrocyte (A); glial filaments (F); inner limiting membrane (ILM); Müller cells (M); vitreous humor (V); G2 (fluvastatin)].

In G1, some astrocytes (Fig 10B1) and Müller cells (Fig 10B2) showed necrotic features. The best-preserved macroglial cells were reactive, with abundant organelles, glial filaments, and clumps of electrodense material. In G2, astrocytes (Fig 10C1) and Müller cells (Fig 10C2) showed the aforementioned signs of glial reactivation. Only a few astrocytes of the glial tufs displayed necrotic changes.

#### Retinal capillaries

**Fig 11:**
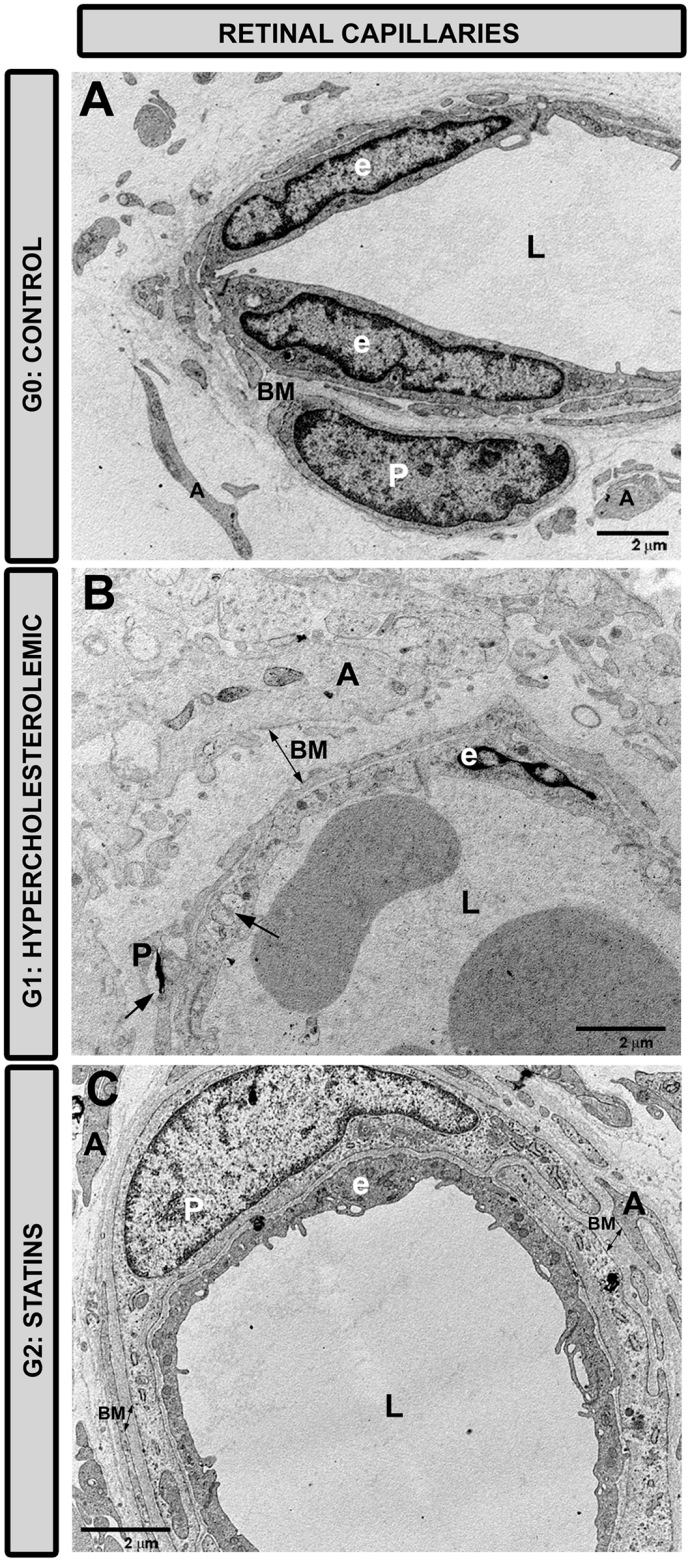
Transmission electron microscopy of retinal capillaries. A: G0 control; B: G1 hypercholesterolemic; C: G2 statins. In G2 (C) the basal membrane was thinner than in G1 (B), being more similar to that in G0 (A). The necrotic features (arrows) observed in the endothelial cells and pericytes in G1 (B) were not found in G2 (C) [basal membrane (BM); endothelial cell (e); vascular lumen (L); pericyte (P); G2 (pravastatin)].

In G1 (Fig 11B) the basal membrane of the capillaries in the NFL and in the vitreous humor proved thicker than in control (Fig 11A) and G2 (Fig 11C). Endothelial cells and pericytes in G1 (Fig 11B) showed the initial stages of necrosis, but these features were absent in G2 (Fig 11C). The vascular lumen appeared normal in both G1 and G2 (Fig 11B and 11C).

### Immunohistochemical Study of the Macroglia in Retinal Whole-Mounts

#### Müller Cells

**Fig 12:**
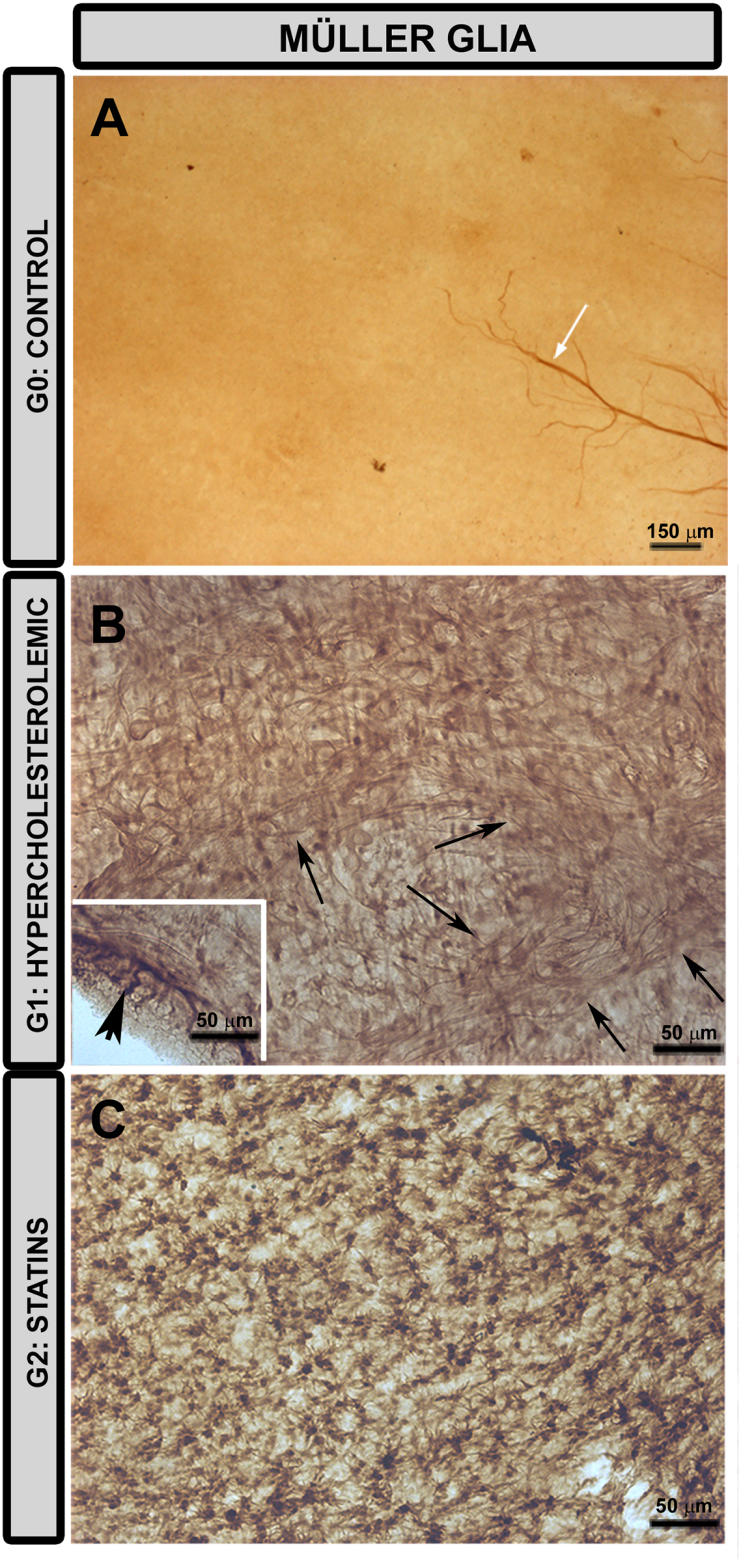
Changes in retinal Müller glia. **Retinal whole-mount. Immunohistochemistry anti-GFAP.** In G0 (A) astrocyte associated with the nerve-fiber bundles were GFAP+ (white arrow); however, immunostaining for GFAP in Müller cells was negative. In G1 (B) and G2 (C) Müller cells were GFAP+. The pressure exerted by the cover slip on the whole-mount caused a retinal-like section effect on one edge of the tissue, revealing that Müller cells were GFAP+ throughout the retinal thickness (arrowhead) (B inset). Only in G1 (B) was a glial scar-like structure observed (arrows).

In control animals (G0), GFAP immunostaining in Müller cells proved negative (Fig 12A), whereas in G1 and G2 (Fig 12B and 12C) the Müller cells were GFAP+. The pressure exerted by the cover glass on the retinal whole-mount caused a retinal-like section effect in some retinal borders in which GFAP+ Müller cells were visible throughout the retina (Fig 12B inset). In G1 and G2 retinas, outside the MNFR where the astrocytes were absent, Müller cells were easier to recognize. These cells formed GFAP+ glial scar-like structures in G1 (Fig 12B) but not in G2 (Fig 12C).

#### Astrocytes

**Fig 13:**
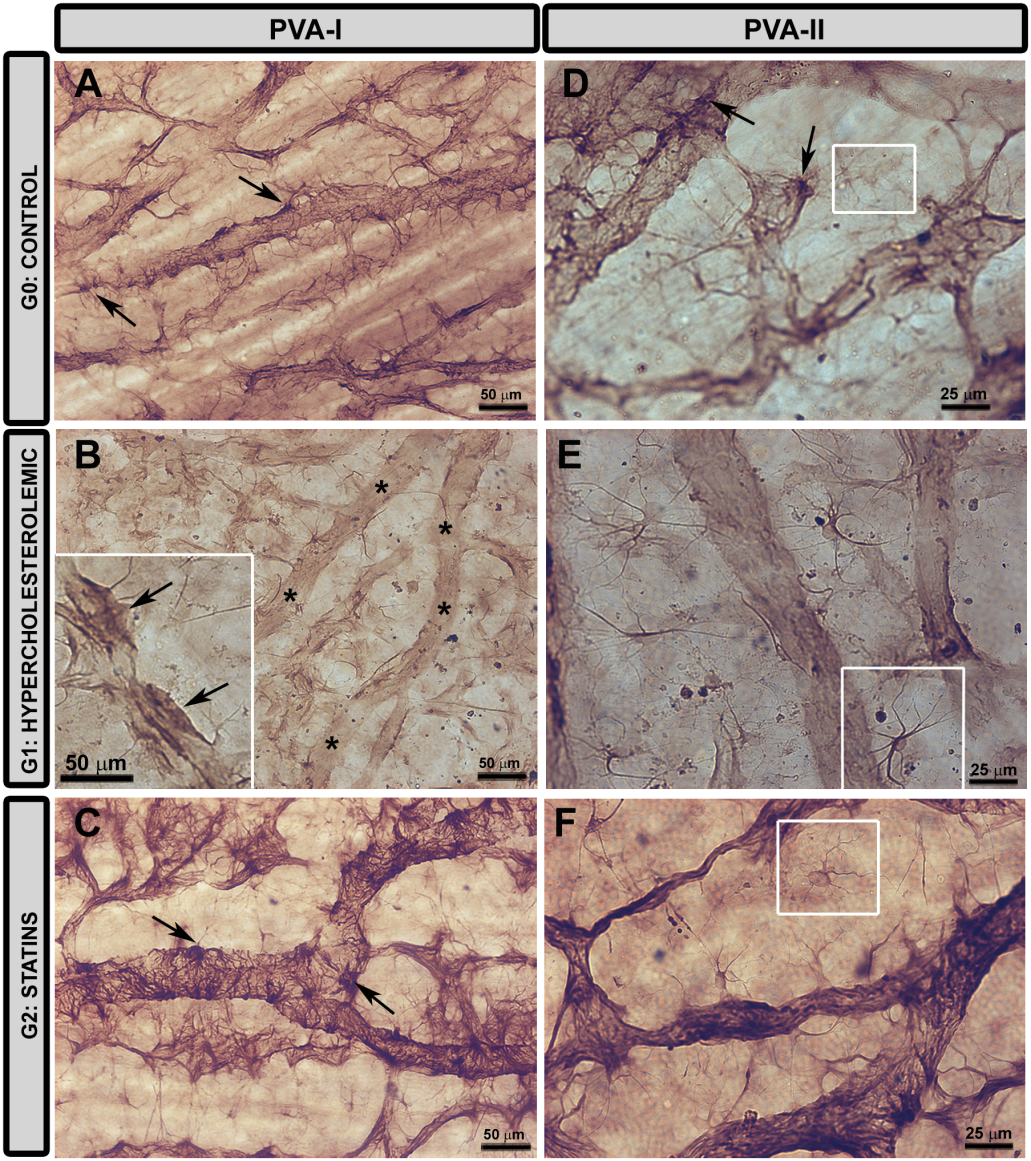
Changes in type I and type II perivascular astrocytes. **Retinal whole-mount. Immunohistochemistry anti-GFAP.** In G0 (A) the soma of PVA-I (arrow) could be easily differentiated from processes. In G1 (B) PVA-I were reactive and soma could not be differentiated from processes (inset). There were large areas free of this astroglial type in many retinal vessels (*). In G2 (C) PVA-I were reactive and somas could be distinguished from processes (arrow). PVA-I processes were so abundant that they almost covered the vessel walls. [type I perivascular astrocyte (PVA-I)]. In G0 (D) GFAP immunostaining in PVA-II (white square) was less intense than in PVA-I (arrow). G1 (E) contained numerous PVA-II in the vicinity of vessels devoid of PVA-I, and the intensity of the GFAP immunoreaction was greater than in G0 (D). In G2 (F), GFAP immunoreaction in PVA-II was lower than in G1 (E) but slightly greater than in G0 (D). In addition, PVA-II morphology in G2 resembled more G0 than G1. [type I perivascular astrocyte (PVA-I); type II perivascular astrocyte (PVA-II)].

**Fig 14:**
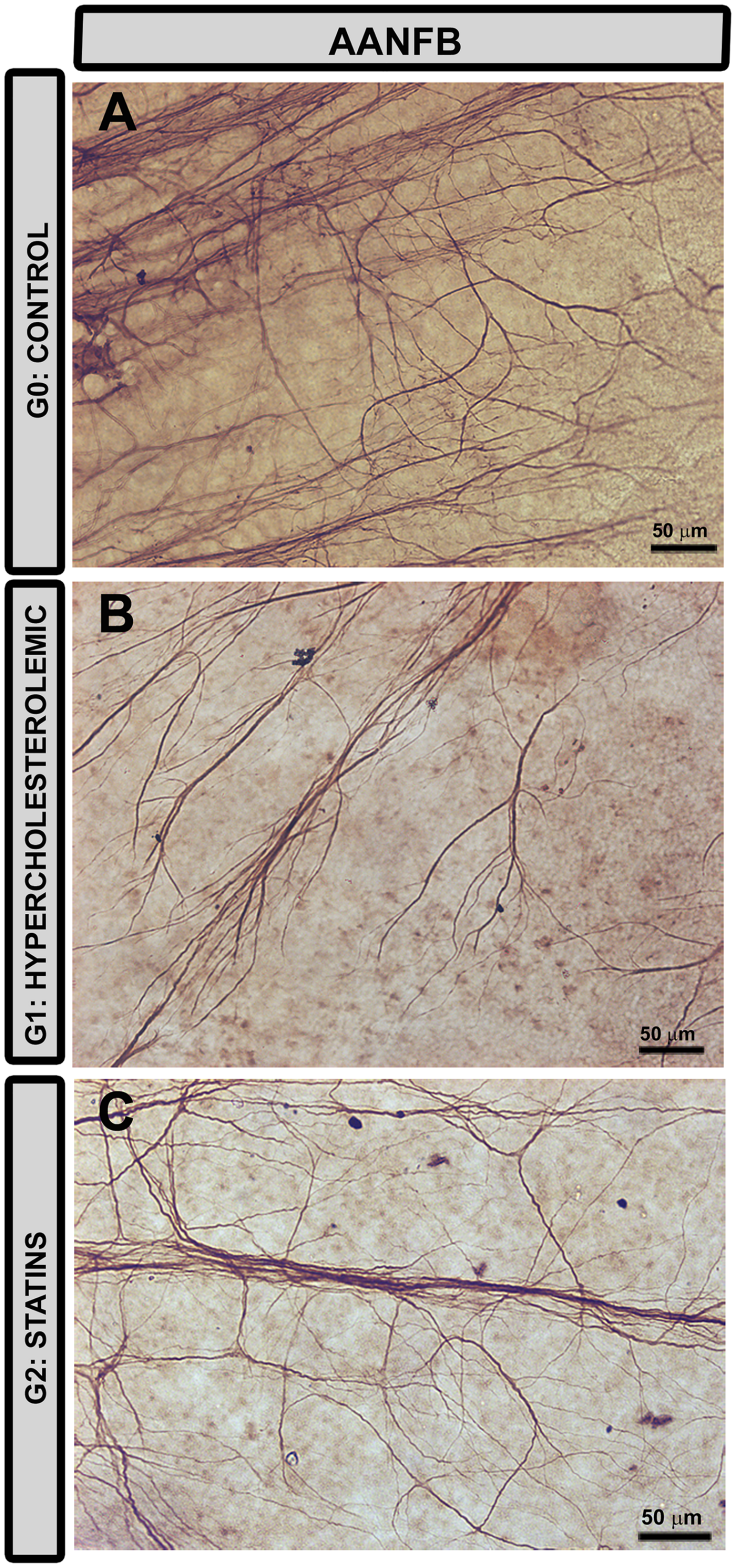
Changes in retinal astrocytes associated with the nerve-fiber bundles. **Retinal whole-mount. Immunohistochemistry anti-GFAP.** In comparison with G0 (A) astrocytes associated with the nerve-fiber bundles in G1 (B) and G2 (C) had thicker cell bodies and processes. [AANFB: astrocytes associated with the nerve-fiber bundles].

In G0, two groups of astrocytes were found: perivascular astrocytes (PVA) (Fig 13A and 13D); and astrocytes associated with the nerve bundles (AANFB) (Fig 14A). The former group contained two types of PVA, PVA-I (Fig 13A) and PVA-II (Fig 13D), as described in the material and method section elsewhere.

Both in G1 (Fig 13B) and G2 (Fig 13C), GFAP PVA-I were reactive. In G1 the vascular walls showed a strong brown-colored reaction due to the presence of thick PVA-I, in which it was not possible to differentiate the soma from the processes (Fig 13B inset). In many retinal vessels (mainly in the medium-sized vessels) there were large areas where this astroglial type had disappeared (Fig 13B). In G2 (Fig 13C), PVA-I were thicker than in G0 (Fig 13A), although, the soma and processes of these cells could be easily distinguished, unlike those from G1. PVA-I had many processes which, in some areas, covered the entire vessel wall (Fig 13C).

In comparison to G0 (Fig 13D), PVA-II in G1 (Fig 13E) had: i) intense GFAP+ immunostaining; ii) a more robust cell body; and iii) longer and thicker processes which made contact with neighboring astrocytes and vascular walls. In G1, PVA-II cells were found on and among the vessels, particularly in those where PVA-I had disappeared (Fig 13E). In G2 (Fig 13F), PVA-II slightly increased in GFAP+ expression as compared to G0 (Fig 13D), though less than in G1 (Fig 13E). In addition, the morphological appearance of PVA-II was similar to that of G0.

In G1 (Fig 14B) and G2 (Fig 14C), AANFB had thicker cell bodies and processes than in G0 (Fig 14A).

#### Retinal area occupied by AANFB

**Fig 15:**
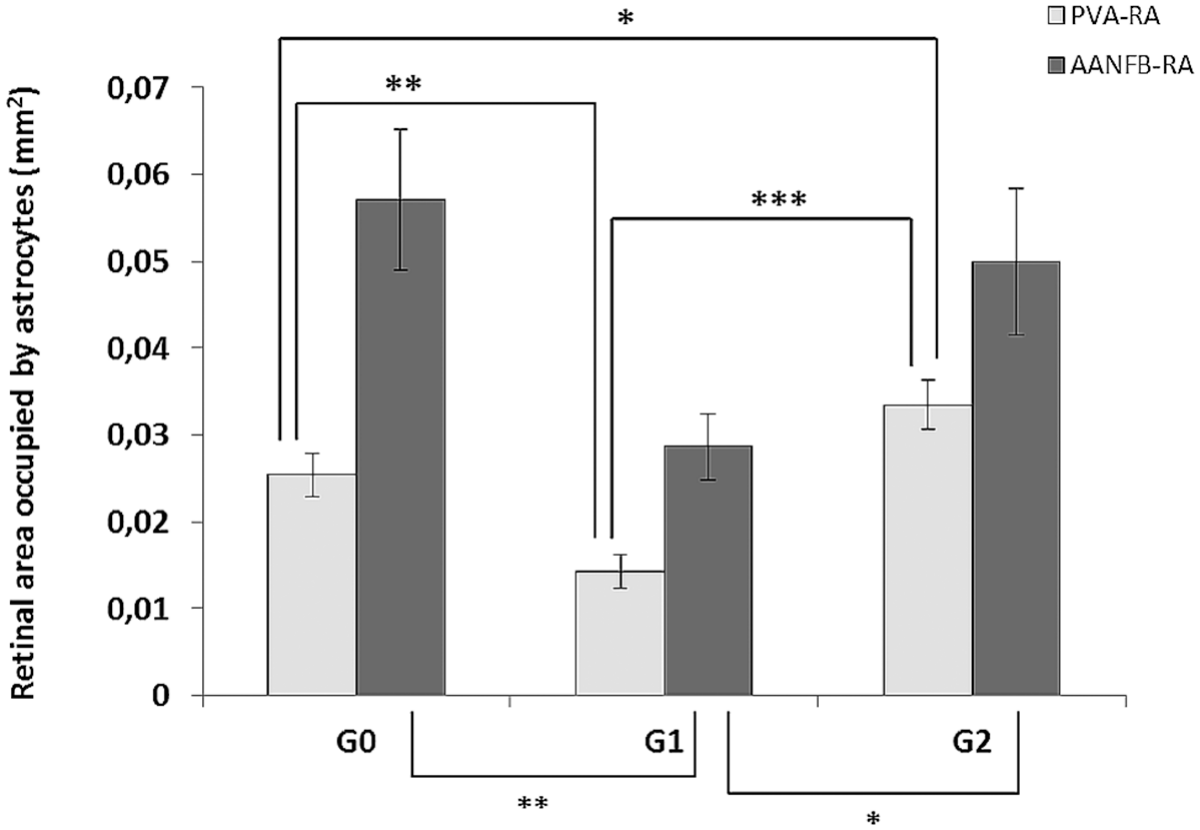
GFAP-labeled retinal area occupied by astrocytes. **Comparison of the three study groups.** The GFAP-labeled retinal area occupied by perivascular astrocytes (PVA-RA) in G1 (hypercholesterolemic untreated rabbits) was significantly decreased in comparison with G0 (control). By contrast, in G2 (hypercholesterolemic rabbits treated with a low dose of statins), the PVA-RA was significantly increased in comparison with both G0 and G1. *** p<0.001 vs. G1; ** p<0.01 vs. G0; * p<0.05 vs. G0. The GFAP-labeled retinal area occupied by astrocytes associated with the nerve-fiber bundles (AANFB-RA) was significantly decreased in G1 (hypercholesterolemic untreated rabbits) in comparison with G0 (control). The comparison of G1 vs. G2 revealed that the AANFB-RA in G2 was significantly larger than in G1. ** p<0.01 vs. G0; * p< 0.05 vs. G1. Each bar represents the mean ± SD. ANOVA with Bonferroni test. ANOVA, analysis of variance.

The comparison of the GFAP+ retinal area occupied by AANFB (GFAP+ AANFB-RA) indicated that all the study groups differed from each other (p<0.01; ANOVA). In comparison to G0 (0.057±0.008 mm^2^), GFAP+ AANFB-RA in G1 (0.029 ±0.004 mm^2^) was significantly reduced (p<0.01 ANOVA with Bonferroni). In addition, in G1 the GFAP+ AANFB-RA was statistically reduced in comparison with G2 (0.050±0.008 mm^2^; p<0.05 ANOVA with Bonferroni) (Fig 15).

#### Retinal area occupied by PVA

The comparison of the GFAP+ retinal area occupied by PVA-I and PVA-II (GFAP+ PVA-RA) showed that all the study groups differed from each other (p<0.001; ANOVA). In comparison to G0 (0.025±0.003 mm^2^), GFAP+ PVA-RA in G1 (0.014±0.002 mm^2^) was significantly reduced (p<0.01 ANOVA with Bonferroni). However, GFAP+ PVA-RA in G2 (0.033 ± 0.003 mm^2^) significantly increased in comparison with both G0 and G1 (p<0.001 and p<0.05, respectively, ANOVA with Bonferroni) (Fig 15).

## Discussion

The New-Zealand rabbit is being used as a model of experimental hypercholesterolemia because, among other characteristics [37–43] atheromateous lesions developed by these animals are similar to those in humans [44]. It bears mentioning that, in this experimental model, lipid deposits also occur in ocular tissues [42,45,46]. Previously, we have reported that high cholesterol levels induce chronic ischemia in the choroid and retina of New-Zealand hypercholesterolemic rabbits [16,18]. Such ischemia was a consequence of the lipid deposit found in the suprachoroidea, vascular endothelium, and Bruch’s membrane (BM). Also, previous work in our laboratory (using the same animals as analyzed in the present study) has demonstrated that a non-lipid-lowering dose of statins improved the damage of the choroidal vessels and BM, as revealed by electron microscopy [33]. Specifically, treatment with fluvastatin sodium and pravastatin sodium at a non-lipid-lowering dose (2mg/Kg/day): i) prevents progressive atherosclerosis in choroidal vessels; ii) preserves the ultrastructural characteristics of vascular smooth-muscle cells and endothelial cells, presumably maintaining the endothelium-depending relaxation and thus diminishing ischemia; and iii) causes the disappearance of lipids in BM [33]. All these features presumably improve oxygen and nutrient transport to the retina [16–18].

Early alterations in the sensory rabbit retina can be secondary to a hypercholesterolemic diet [16,17,47]. However, to the best of our knowledge, no works available demonstrate that treating these animals with a statin dose insufficient to normalize plasma lipid levels (2mg/Kg/day) [27,48] can preserve the retinal ultrastructure. Therefore, in the present study, we offer an in-depth report on the effects of non-lipid-lowering doses of fluvastatin and pravastatin on the retina in hypercholesterolemic rabbits, specifically assessing: i) ultrastructural changes of the retinal layers, retinal vessels, and retinal macroglia; ii) retinal-layer thickness; iii) GFAP+ area occupied by retinal astrocytes and; iv) immunohistochemical changes of retinal macroglia.

Statins, selective inhibitors of 3-hydroxy-3-methyl-glutaryl-CoA reductase, can lower serum-cholesterol levels in humans and animals by depressing cholesterol biosynthesis [49,50]. Apart from their beneficial effects as lipid-lowering drugs, statins have additional valuable pleiotropic properties [27–30,51], which have been reported in hypercholesterolemic rabbits at a dose inadequate to reduce blood-cholesterol levels [27,32]. Recently, it has been reported that in human AMD patients high-dose statints may result in drusen regression [52]. However, studies on statins and AMD are inconclusive to date because such studies have considered all hydrophilic and lipophilic statins together, so that an assessment of a local ocular effect is not possible [53]. The reason for using two different statins in the present study was not to compare the intensity of their respective effects, but to check whether both lipophilic (fluvastatin) and hydrophilic (pravastatin) statins with different pharmacodynamic properties [33] were able to protect the retina. In mice, both lipophilic (simvastatin) and hydrophilic (pravastatin) statins reportedly cross the blood-brain-barrier (BBB) [31]. Lipophilic statins administered as lactone forms cross the BBB directly. However, the mechanisms proposed for actively transporting hydrophilic statins into the brain are organic anion transporters [54] and by the monocarboxylic acid transporter [55]. In the cerebral cortex of mouse, both hydrophilic as well as lipophilic statins pleiotropically affected neuroprotective gene expression [31]. This could explain why in the present study we found that both statin types caused similar effects on the retina. Statins could contribute to neuroprotection through at least three different pleiotropic effects: i) reduction of the oxidative damage by diminishing the production of reactive oxygen species [56,57]; ii) improvement of vascular function by controlling nitric oxide production [22]. Statins improved blood flow even further by suppressing the expression of the vasoconstrictive peptide endothelin-1 [58,59] and; iii) modulation of the immune response by reducing cytokine-induced expression of costimulatory molecules on immune cells and endothelium and expression MHC-II class molecules. In addition, statins interact with adhesion molecules and modulate their expression [60].

The retina is a demanding tissue in terms of cholesterol use. Retinal cholesterol comes from two different sources: local synthesis and extra-retinal supplies. The blood-retinal barrier, in contrast to BBB, is capable of cholesterol uptake from the circulation via a lipoprotein-based/receptor-mediated mechanism [2]. As established in studies using systemic injection of fluorescently-labeled lipoprotein particles and subsequent fluorescence imaging of retinal cross section, cholesterol from the systemic circulation can cross the RPE [3]. Thus, blood-borne lipoproteins reach the RPE as well as the neuroretinal layers [61]. In accordance with a previous study [16], we found here that, in hypercholesterolemic rabbits, RPE cells showed intracellular droplets of lipids. These lipid accumulations may occur because RPE expresses a variety of lipoprotein-specific receptors (LDLR, SR-BI, SR-BII) and scavenger receptors (CD36) that enable the recognition of circulating lipoproteins by RPE and delivery to the retina [1,10]. The accumulation of lipids between the inner collagenous layer and the basal lamina of the BM (as seen in many older human eyes) participates in creating a physical barrier, called the lipid wall, which may limit the exchanges between the choriocapillaris and the RPE [10,62,63]. This fact is believed to result in a lower nutrient intake by the neural retina, compromising retinal function [2,16,33].

Brain cholesterol is synthesized *de novo* in neurons and astrocytes [4,64,65] while, in the retina, synthesis occurs not only in these cells but also in the Müller glia [66] and RPE [24]. It has been reported that, in mice, neither a high-cholesterol diet nor orally administered simvastatin significantly affected retinal expression of the major cholesterol- and vision-related genes and that these treatments had only a modest impact on the cholesterol content in the retina [67,68]. This behavior is essential for the retina, because cholesterol is a lipid source and is required to build the vast membrane surfaces of the axons, dendrites, and synapses [69,70].

Our study demonstrates that the appearance of the retina was better preserved in hypercholesterolemic rabbits receiving low-dose statins (G2) than in hypercholesterolemic rabbits without treatment (G1). The preservation of the retinal ultrastructure found in G2 could be due to the pleiotropic effects of statins. One such effect could be related to ischemia reduction. In hypercholesterolemic rabbits, the use of the same statins at the same dose as used in the present work have been found to help preserve endothelial cells in the choroidal vessels [33]. A similar effect was noted in the retinal vessels in G2 rabbits of the current study, suggesting that an endothelium-dependent relaxation is maintained, thus reducing retinal ischemia. Statins can reduce the production of reactive oxygen species by inhibiting the activation and assembly of the NADPH-complex [56,57], by controlling nitric oxide production, and possibly by reducing the inflammatory response [22]. According to this scenario, the decreased cell necrosis detected in G2 could account for the diminished cell edema in G2, and may partly explain why the thickness of the different retinal layers in G2 was significantly reduced in comparison to G1.

The presence of well-known lipid transport proteins involved in HDL particles maturation in the photoreceptor layer has been reported [61]. These molecules are involved into the uptake and turnover of normal (non-oxidized) lipid species in retinal cells and may also facilitate the removal of oxidized lipids, especially those arising in the membrane of the outer segment of the photoreceptors [61]. In the photoreceptor layer, the swollen and degenerated mitochondria of the ellipsoid in G1 lead us to postulate that the aforementioned lipid transport system is impaired in hypercholesterolemic rabbits. By contrast, many well-preserved mitochondria were found in the photoreceptor layer in G2. Such mitochondrial preservation was found in the rest of retinal layers in G2. Notably, in G2 this preservation was accompanied by fewer necrotic and apoptotic features in these animals. It has been proposed that apoptosis can be intensified by the release of apoptogenic factors after the opening of the mitochondrial permeability transition pores, while persistent opening leads to necrotic cell death [71]. The ability of the mitochondrial state to induce apoptosis or necrosis could help explain why the better the mitochondrial preservation (as in animals treated with low statin doses) the lesser the retinal cell death. This could in turn account for the fact that, in G2, features of cell death in the nuclear layers were reduced in comparison with non-treated animals.

Statins can alter the expression of specific genes associated with apoptosis [31], thus protecting the neurons from ischemic insults [72,73]. They can also reduce apoptotic transduction signals induced by hypoxic ischemia, lowering the caspase-3 activation, the most important protease in the apoptotic pathway [74]. The above effects of statins in apoptosis could account for the reduced neuronal death by apoptosis found in our low-dose statin-treated group.

The analysis of the plexiform layers revealed that synaptic complexes were better preserved in G2 in which numerous synaptic vesicles, the synaptic ribbon, and arciform density were visible. The most abundant neurotransmitter in the retina is glutamate and its accumulation promotes excitotoxic neuronal death. Statins modulate glutamate receptors as well as transporter localization and function, in addition to glutamate metabolism via glutamate synthase activity, contributing to the antiexcitotoxic properties of statins [64,75–77]. In addition, statins stimulate BDNF expression [78,79]. BDNF regulates synaptic plasticity and appears to protect the synaptic molecular complex [79].

A hypercholesterolemic diet in rabbits reportedly may induce early changes in the sensory retina, with loss of RGC number [16,47]. In our G1, all ganglion cells were necrotic. In G2, most of the ganglion cells presented normal features and the axons in the nerve-fiber layer were better preserved than in G1. *In vivo* statin treatment has been shown to protect RGC after ischemia-reperfusion [80,81]. It seems that statins exert neuroprotection by modulating Bcl-2, BAX, BDNF, and heat-shock protein expression as well as activating of the Akt, Wnt, and ERK signaling pathway, which are all known mediators of RGC survival [82].

Astrocytes and Müller cells protect the CNS from damage through a process called reactive gliosis, which is triggered by polyetiological insults [83–85]. The upregulation of GFAP, a commonly used marker for reactive Müller cells, is so sensitive that it can serve as an indicator of retinal stress, retinal injury, and Müller cell activation [86]. The retinal stress secondary to the ischemic insult induced by hypercholesterolemia was manifested as reactive Müller cells in both the G1 and G2 retinas. However, in the low-dose statin-treated animals the glial scar-like structures formed by Müller cells in G1 were not detected.

The reduction of the retinal area occupied by AANFB and PVA in G1 was not detected in G2 and, in comparison with control in G2, the retinal area occupied by the PVA increased. Such findings could be explained by: i) the reduction in astroglial necrosis in G2 in comparison with G1 observed with TEM and, ii) the astroglial hypertrophy that accompanies gliosis.

After different types of injuries, astrogliosis is a major event in CNS cell repair. The neuroinflammatory environment brought about by astrogliosis is secondary to the activation of NF-кB by reactive astrocytes that produce TNF-α and nitric oxide [87,88] In addition, reactive astrocytes express several cell-adhesion molecules and chemokines which facilitate the infiltration of inflammatory cells to the injury site [89]. This scenario may be associated with neuronal damage. Statins suppress neuroinflammation by blocking the activation of astroglial NF-кB [89]. This mechanism could at least partly be the reason why neuronal damage was lowest in G2.

In G1 retinal whole-mounts, it was observed that PVA-I disappeared from blood vessels in some retinal areas. This occurrence could have a functional implication, given that astrocytes and Müller cells: i) induce blood-retinal barrier properties in the retinal capillaries [90,91] by releasing substances that stabilize tight junctions between endothelial vascular cells [90], securing immune privilege to protect neurons from the damage of an inflammatory immune response [92] and; ii) help regulate blood flow [93] in response to changes in neuronal activity [94] by secreting a number of molecules, such as prostaglandins, nitric oxide, and arachidonic acid [95,96]. It bears noting that, in G2 retinas, no loss of PVA-I was detected, suggesting that BRB and blood flow would be better maintained, perhaps accounting for the better retinal preservation and lower cell death found in low-dose statin-treated animals.

A major consequence of the astroglial decrease (observed as a reduction of AANFB-RA and PVA-RA) in G1 could be related to the fact that these cells are an active partner of neurons by maintaining cholesterol synthesis and removal [1]. In the retina, Müller cells and astrocytes participate in the metabolism (supplying heterogeneous lipoprotein particles and ApoE) and transport of cholesterol, an essential source of lipids for the maintenance of the neuronal cell membranes [17,66,69,97], making neurons susceptible to alteration in long-term hypercholesterolemia. The Apo E supply to neurons by the better-preserved astroglial population observed in the retinas of rabbits treated with low-dose statins could contribute to the maintenance of the retinal neurons found in this group of animals. Another mechanism involved in astrocyte neuroprotection in G2 could be the antioxidant capacity that follows the activation of these cells. Thus, by means of enzymatic and non-enzymatic antioxidant defenses reactive astrocytes protect neurons from free radicals [98]. The fact that in G2 the astroglial population was preserved and reactive moves us to postulate that their antioxidant capacity was maintained.

## Conclusion

In conclusion, this study, using the New Zealand rabbit model of hypercholesterolemia, is the first to report that a non-lipid-lowering dose (2mg/Kg/day) of fluvastatin sodium or pravastatin sodium may prevent retinal degeneration, possibly through pleiotropic effects. This conjecture is based on the following observations in statin-treated animals in comparison with non-treated ones: i) the structure of the retina in semi-thin sections looked similar to control; ii) the ultrastructure in all retinal layers was better preserved; iii) the degeneration in the nuclear layers decreased; iv) the hydropic degeneration in the plexiform and nerve-fiber layers diminished; v) the anoxic edema secondary to hypercholesterolemia was reduced in all retinal layers; and vi) retinal astrocytes and vascular structures were preserved. Our findings indicate that low doses of statins can prevent retinal degeneration, acting on retinal macroglia, neurons and retinal vessels, despite that hypercholesterolemia remained unchanged. Thus, the pleiotropic effects of the statins may help safeguard the retinal ultrastructure.
